# An Isoflavone Synthase Gene in *Arachis hypogea* Responds to *Phoma arachidicola* Infection Causing Web Blotch

**DOI:** 10.3390/plants13212948

**Published:** 2024-10-22

**Authors:** Xinying Song, Ying Li, Xia Zhang, Tom Hsiang, Manlin Xu, Zhiqing Guo, Kang He, Jing Yu

**Affiliations:** 1 Shandong Peanut Research Institute, Qingdao 266100, China; songxinying88@126.com (X.S.); li1989ying0921@163.com (Y.L.); zhangxia2259@126.com (X.Z.); xumanlin@126.com (M.X.); zhiqingivy2011@hotmail.com (Z.G.); hekang@qibebt.ac.cn (K.H.); 2 School of Environmental Sciences, University of Guelph, Guelph, ON N1G 2W1, Canada; thsiang@uoguelph.ca

**Keywords:** *Arachis hypogaea*, web blotch, isoflavone synthase gene, *Phoma arachidicola*

## Abstract

Peanut web blotch is an important leaf disease caused by *Phoma arachidicola*, which seriously affects the quality and yield of peanuts. However, the molecular mechanisms of peanut resistance to peanut web blotch are not well understood. In this study, a transcriptome analysis of the interaction between peanut (*Arachis hypogaea*) and *P. arachidicola* revealed that total 2989 (779 up- and 2210 down-regulated) genes were all differentially expressed in peanut leaves infected by *P. arachidicola* at 7, 14, 21 days post inoculation. The pathways that were strongly differentially expressed were the flavone or isoflavone biosynthesis pathways. In addition, two 2-hydroxy isoflavanone synthase genes, IFS1 and IFS2, were strongly induced by *P. arachidicola* infection. Overexpression of the two genes enhanced resistance to *Phytophthora parasitica* in *Nicotiana benthamiana*. Knockout of *AhIFS* genes in peanut reduced disease resistance to *P. arachidicola*. These findings demonstrated that *AhIFS* genes play key roles in peanut resistance to *P. arachidicola* infection. Promoter analysis of the two *AhIFS* genes showed several defense-related cis-elements distributed in the promoter region. This study improves our understanding of the molecular mechanisms behind resistance of peanut infection by *P. arachidicola*, and provides important information that could be used to undertake greater detailed characterization of web blotch resistance genes in peanut.

## 1. Introduction

Peanut (*Arachis hypogaea*) is an important economic crop cultivated in many subtropical and tropical areas of the world. In North and South America, Africa, and Asia, peanuts are an important source of oil and protein [1], and are widely grown in semi-arid tropical areas. China makes the greatest contribution to global production (41.6%) followed by India (12.5%) [2].

Peanut web blotch is a significant foliar disease of peanut, and was first reported in Texas, USA, in 1972 [3]. Typical symptoms are net-like bronzing with large light brown blotches on infected peanut leaves. Subsequently, the disease was also documented in Russia, Argentina, South Africa, China, and Spain [4]. There has been some debate as to whether the pathogen responsible for peanut web blotch is species of *Phoma* or of *Ascochyta* [3,5]. Currently, *Phoma arachidicola* Marasas Pauer & Boerema is widely recognized to be the causal agent of peanut web blotch [3,6]. The disease leads to extensive leaf drop during the later stages of peanut growth, and significantly reduces yields by 10% to 20%, with severe cases resulting in reductions of over 30% [7]. Consequently, it is an important issue in regions where peanuts are cultivated.

Despite its economic importance, research on this disease has been limited compared to other major foliar diseases affecting peanuts. A draft genome sequence previously was published for a *P. arachidicola* isolate named Wb2 [8]. The draft genome size was 34.11Mb and contained over 37,000 open reading frames (ORFs) with a G+C content of over 49%. They found that resistance to web blotch was controlled by three major genes as well as several minor genes, and uncovered one quantitative trait locus on a linkage group [9]. Previous research on peanut web blotch focused primarily on classifying the taxonomy of the pathogen, its molecular biology, chemical control, and epidemiology [4,5,10]. Currently, there is a paucity of studies on genes associated with peanut web blotch resistance, and even fewer reports on the underlying resistance mechanism. This lack of information significantly hampers progress in breeding for peanut web blotch resistance.

To analyze the molecular mechanism of peanut resistance to *P. arachidicola* infection, a peanut cultivar Huayu 23 that showed susceptibility to *P. arachidicola* was inoculated with the conidial suspension of *P. arachidicola* and then subjected to RNA-seq analyses. The results showed that flavonoid and isoflavonoid biosynthesis genes were up-regulated during *P. arachidicola* infection, which suggested they had a potential role in resistance to this pathogen. However, there have been no studies on the involvement of these two genes in peanut resistance.

The isoflavonoids are a diverse group of secondary metabolites. They are primarily found in leguminous plants, and are synthesized by the phenylpropanoid pathway. Isoflavone synthase (IFS) is a major enzyme in synthesis of isoflavonoids, and has a substrate in common with flavone synthase II and flavanone-3-hydroxylase [11]. Isoflavones also play crucial roles in plant-microbial interactions where they act as chemoattractants for rhizobia, and induce the expression of genes associated with nitrogen-fixing root nodule formation [12,13]. Additionally, isoflavones function as defense compounds during disease resistance response [14]. Extensive research has been done on regulatory mechanisms controlling isoflavone biosynthesis via the phenylpropanoid pathway [11]. The transcriptional levels of genes associated with isoflavone biosynthes are directly regulated by the ectopic expression of plant transcription factors, such as MYB, basic helix loop helix (bHLH), basic leucine zipper protein (bZIP), WRKY, MADS-box, and WD40 proteins [15,16,17,18].

To verify the anti-*P. arachidicola* infection function of two isoflavone synthesis genes in peanut, *AhIFS1* and *AhIFS2* were cloned and further characterized. The expression of them gradually increased with infection time, which meant that their expressions were continuously up-regulated as time progressed. Overexpression of the two genes enhanced resistance levels to *Phytophthora parasitica* in *Nicotiana benthamiana* leaves and knockout of the two genes reduced disease resistance to *P. arachidicola* in peanut. We hypothesized that when *P. arachidicola* infects peanut leaves, isoflavone synthesis gene are up-regulated, and a large amount of isoflavones are synthesized, which increases peanut resistance. This process may be regulated by one or more promoters. The objectives of this work were to explore the molecular mechanism of peanut resistance to anti-*P. arachidicola*. They also enhance understanding about the interaction mechanisms between *P. arachidicola* and peanut plants and provide a theoretical basis for breeding resistance to peanut web blotch.

## 2. Results

### 2.1. Peanut Responses to Inoculation with P. arachidicola

**Figure 1:**
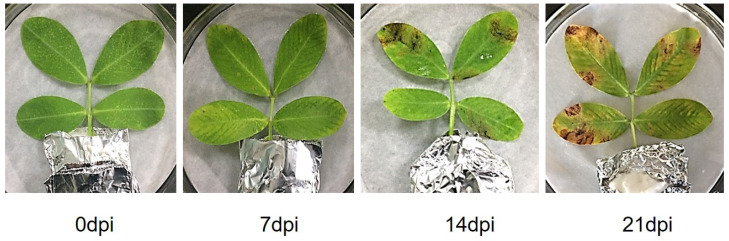
Symptoms on peanut leaves inoculated with *P. arachidicola* conidial suspensions at 0, 7, 14, and 21 dpi.

The pattern of occurrence and development of peanut web blotch were investigated by inoculating detached peanut leaves with *P. arachidicola* conidial suspensions and then observing the reactions that occurred. The results showed that by 7 days post infection (dpi), the leaf veins were green and yellow, and needle eye lesions appeared on the leaves. Typical irregular large disease spots appeared on the leaves at 14 dpi (Figure 1). At 21 dpi, there was a yellow halo around the disease spots, and multiple disease spots joined together to form larger disease spots, which in some cases covered the entire leaf.

### 2.2. RNA Sequencing and Novel Gene Prediction

Peanut leaves that had been inoculated with *P. arachidicola* conidial suspensions were removed for analysis at 0, 7, 14, and 21 dpi. There were three replicates and 12 samples subjected to RNA sequencing using the Illumina HiSeq platform (Illumina, San Diego, CA, USA). Each sample produced an average of 6.86 Gb of data and the average ratios of the samples to the genome and the gene set were 92.54% and 83.89%, respectively. A total of 91,823 genes were predicted. The RNA reads were assembled to yield this very large number of putative transcripts which likely contains both genes from peanuts and from the pathogen, among which 42,672 had recognized homologs to peanut genes and a further 5862 were long-chain non-coding RNAs (Table S1).

### 2.3. Differentially Expressed Transcripts

**Figure 2:**
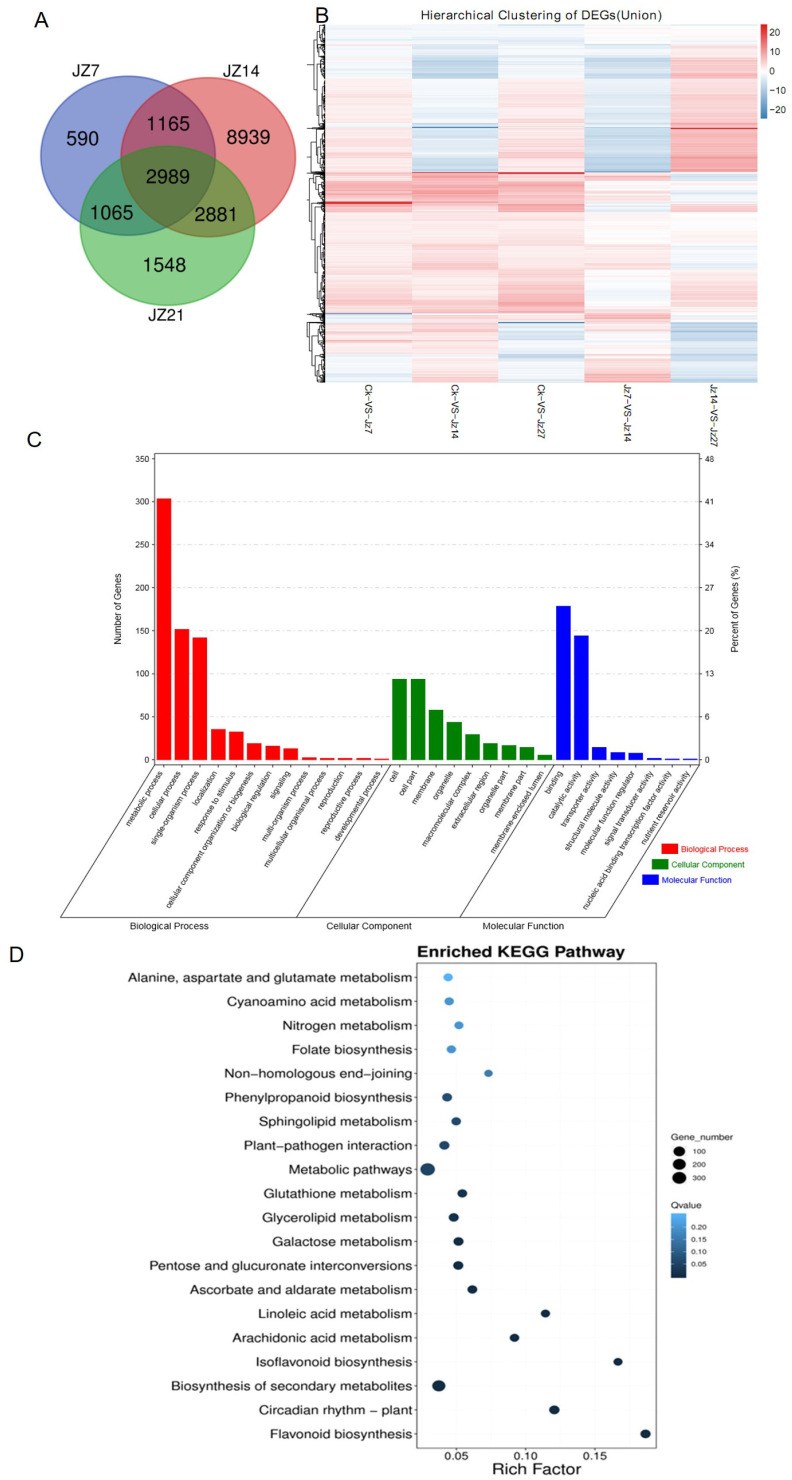
Gene characterization and analysis for differentially expressed genes (DEGs). (**A**) Venn dialog of expressed genes from genome analysis showing the number of expressed genes in peanut leaves that were infected by *P. arachidicola* at 7 (JZ7), 14 (JZ14), 21 (JZ21) dpi. The numbers represent the counts of common or uniquely expressed genes. (**B**) Heatmap of DEGs. Red represents up−regulated genes; blue represents down−regulated genes. Darker colors represent intensity where darker red colors show the most upregulated and the darker blue are the most downregulated genes. (**C**) Go analysis of gene function classification shows the number of genes involved in biological processes, cell components, and molecular functions, respectively. (**D**) KEGG pathway enrichments of DEGs. The panel showing 20 enriched KEGG terms for DEGs responding to *P. arachidicola* infection. The legend is shown on the picture.

The differentially expressed genes (DEGs) were calculated from three independent biological replicates that were then subjected to a transcriptomic analysis to determine the gene expression change after varying times of *P. arachidicola* infection period. The DEGs were defined as those that had an expression ratio of more than 2 times and where q ≤ 0.05. The number of genes that responded to *P. arachidicola* infection was relatively low at 7 dpi (5,809 DEGs). At 14 dpi which is the mid-phase of infection, when obvious symptoms appear on peanut leaves, the number of genes that responded increased sharply to 15,974 DEGs. However, the number of DEGs decreased again to 8483 at 21 dpi, which is the late stage of infection when severe symptoms appear on peanut leaves. A Venn diagram of the DEGs revealed that 2989 genes responded to *P. arachidicola* infection at the three different time points (7, 14, 21 dpi), indicating that these were involved in the response to *P. arachidicola* infection to different degrees throughout the infection process. A hierarchical clustering method was used to examine the overall expression patterns of the 2989 DEGs (Figure 2A). Most DEGs showed differences in expression levels after *P. arachidicola* infection compared to the control plants (Figure 2B).

### 2.4. Gene Ontology (GO) and KEGG Pathway Enrichment of DEGs

A GO enrichment analysis identified the GO categories for the DEGs. There were more DEGs with GO terms that included biological process (BP) than for cellular component (CC) and molecular function (MF) (Figure 2C, Table S2). The enriched GO terms in each category were also determined and metabolic process-related terms were the most abundant in the BP, MF, and CC categories.

A KEGG analysis of the 2989 DEGs was also performed (Figure 2D) to further analyze the pathways that respond to *P. arachidicola* infection. A substantial proportion of the genes were associated with the metabolic and secondary metabolic biosynthesis pathways, but the Rich factors for the two gene types were low, which meant that the enrichment effect was not significant. Although the flavonoid and isoflavonoid biosynthesis pathway genes were not the most numerous, they did show the greatest enrichment effect, which suggests that they probably play key roles in peanut resistance to *P. arachidicola* infection.

### 2.5. Functional Analysis of the Isoflavanone Synthase Genes

**Figure 3:**
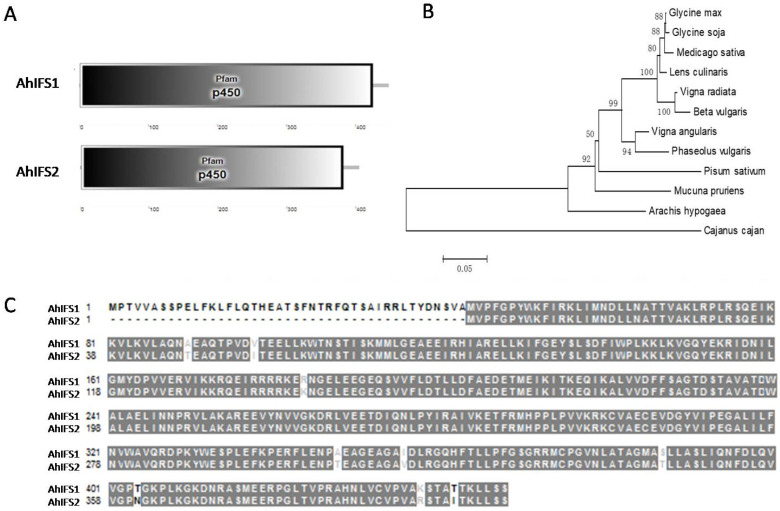
Analysis of IFS protein function. (**A**) Functional domain schematic for the AhIFS proteins (Arahy.1WY37S and Arahy.B6E5H4). The predicted functional domains (http://smart.embl-heidelberg.de/) indicated that both proteins contained P450 domains. (**B**) Phylogenetic tree analysis of the AhIFS orthologs. Close orthologs from *Arachis hypogaea* and other plants, including GmIFS1 (*Glycine max*, NP_001236022.1), GsIFS1 (*Glycine soja*, ACA81484.1), MsIFS1 (*Medicago sativa*, AAF34521.1), LcIFS1 (*Lens culinaris*, AAF34525.1), VaIFS1 (*Vigna angularis*, XP_017422719.1), PsIFS1 (*Pisum sativum*, AAQ10282.2), MpIFS1 (*Mucuna pruriens*, RDX63227.1), CcIFS1 (*Cajanus cajan*, AEQ39026.1). (**C**) AhIFS proteins were used for IFS protein sequence alignment. Amino acid identity of 100% is represented with a black background; >50%, with black text on a gray background; and similar amino acids have white text on a gray background.

To find key genes for peanut resistance to web spot, two 2-hydroxyisoflavanone synthase genes were identified and named *AhIFS1* and *AhIFS2*. The predicted functional domains (http://smart.embl-heidelberg.de/ accessed on 1 June 2020.) indicated that both proteins contained P450 domains (Figure 3A).

A blast analysis of the IFS protein sequences against NCBI (https://blast.ncbi.nlm.nih.gov/Blast.cgi accessed on 12 June 2020.) showed that there were a large number of homologous proteins and these proteins are relatively conservative in different species. A total of 11 homologous sequences from different species were selected to create a phylogenetic tree (Figure 3B) using maximum likelihood method in MEGA6.0 software. The results showed that there was a large genetic distance between the IFS proteins in peanut and those in soybean, alfalfa, and other species, which means that it is impossible to predict whether the IFS protein in peanut has a similar function to the IFS proteins in other species.

A comparison of the IFS1 and IFS2 protein sequences showed that the 43 amino acids at the beginning of IFS1 were not present in IFS2 and only nine amino acids were different in the rest of the sequence (Figure 3C). This shows that IFS1 and IFS2 have a high homology and may have similar functions.

### 2.6. Expression Patterns forAhIFS Genes

**Figure 4:**
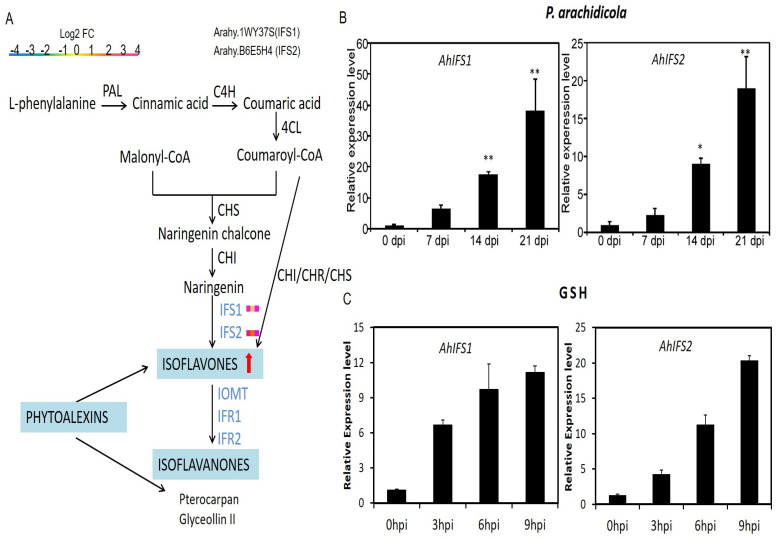
The expression pattern of *AhIFS* genes. (**A**) Flavonoid and isoflavone biosynthesis pathways. Enzymes are indicated in uppercase letters. PAL, phenylalanine ammonia-lyase; C4H, cinnamate 4-hydroxylase; 4CL, 4-coumarate: CoA ligase; CHS, chalcone synthase; CHI, chalcone isomerase; IFS, isoflavone synthase; The expression of IFS genes refers to the expression of genes at three infection time points (7, 14, 21 day) in the transcriptome. (**B**,**C**) Transcriptional expression of *AhIFS* genes at the infection stage. The y- and x-axes represent the relative expression levels and time course for *P. arachidicola* infection, respectively. Mean values and standard deviations (SDs) were obtained from three biological and three technical replicates. Error bars are SDs; time points are 0, 7, 14, and 21 hpi; * and ** indicate that the expression level was significantly and extremely significant different from the control.

By analyzing the transcriptome data, it was found that the two *AhIFS* genes expression level was significantly up-regulated in the three time periods of *P. arachidicola* infection (Figure 4A). The expression patterns of the two IFS genes in peanut leaves (*AhIFS1* and *AhIFS2*, respectively) infected by *P. arachidicola* were detected by qRT-PCR. The analysis showed that *AhIFS1* and *AhIFS2* had the same expression patterns. Expression of the *AhIFS* genes gradually increased with infection time, which meant that their expressions were continuously up-regulated as time progressed (Figure 4B). Previous research has illuminated that glutathione (GSH) therapy can stimulate the production of isoflavonoids [19]. To ascertain whether the two pivotal genes in isoflavone biosynthesis—*AhIFS1* and *AhIFS2*—are activated by GSH, we delved into the expression patterns of AhIFS1 and AhIFS2 in peanut leaves subjected to GSH treatment. Upon exposure to GSH for 3 h, *AhIFS1* and *AhIFS2* exhibited a remarkable 6-fold and 4-fold surge in expression, respectively, compared to the control samples. Upon exposure to GSH for 9 h, these two genes exhibited a remarkable 11-fold and 20-fold surge in expression, respectively. Hence, both AhIFS1 and AhIFS2 exhibited a pronounced upregulation in their expression levels upon GSH elicitation. Notably, the expression patterns of AhIFS1 and AhIFS2 mirrored those observed during *P. arachidicola* infection, as well as paralleled the isoflavone content, thereby indicating a strong correlation between the expression of these genes and the accumulation of isoflavones in peanut. This underscores the pivotal role of AhIFS1 and AhIFS2 in modulating isoflavone biosynthesis in peanut.

### 2.7. Overexpression of AhIFS Genes in N. benthamiana Enhance Its Disease Resistance

**Figure 5:**
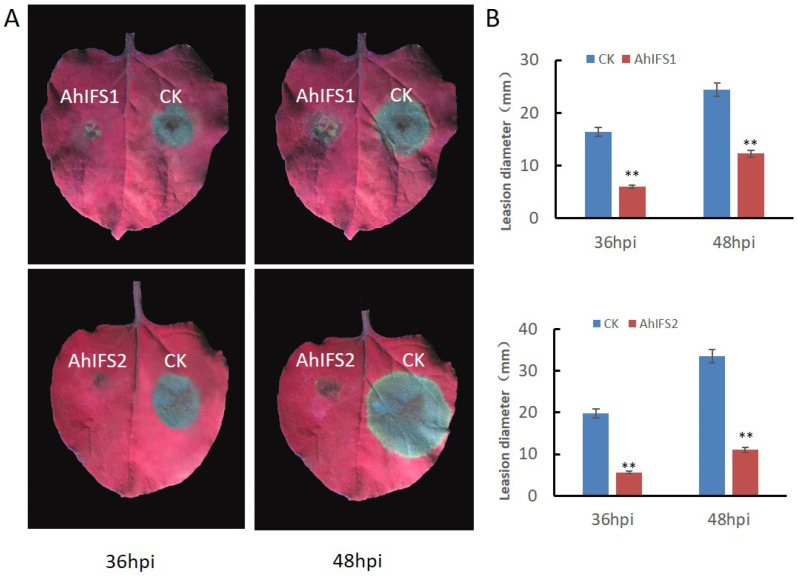
Overexpression of AhIFSs enhances resistance of *N. benthamiana* to *P. parasitica*. (**A**) Transient expression of CK (right) and AhIFSs (left) fusion proteins in *N. benthamiana* leaves mediated by *A. tumefaciens*. The infiltrated leaves (after 48 h) were inoculated with *P. parasitica* zoospores and photographed at 36 h post infection (hpi) and 48 hpi respectively. (**B**) Lesion diameters (mm) of *N. benthamiana* leaves. The data were calculated from three independent biological replicates, with at least eight leaves in each replicate (** *p* < 0.01 when compared with GFP; Dunnett’s test).

The roles of *AhIFS* genes in plant disease resistance were further explored. To do this, the full—length cDNA of these genes was fused with a GFP—tag sequence at the N—terminus, thereby generating the construct pBINPLUS:GFP:AhIFS. A transient expression assay mediated by *Agrobacterium tumefaciens* was carried out to express the GFP:AhIFS fusion protein in the leaves of *N. benthamiana*. The GFP protein alone served as a negative control. After transient expression, the corresponding *N. benthamiana* leaves were inoculated with zoospore suspensions of *Phytophthora parasitica*. The lesion diameters were measured at 36 and 48 h post—infection (hpi). The leaf surface that expressed the AhIFSs developed significantly smaller lesions than the leaf surface that expressed the GFP control (Figure 5). These results showed that the overexpression of AhIFSs strengthened the disease resistance of *N. bethaminiana* against *P. parasitica*.

### 2.8. Knockout of AhIFS Genes Reduces Disease Resistance

**Figure 6:**
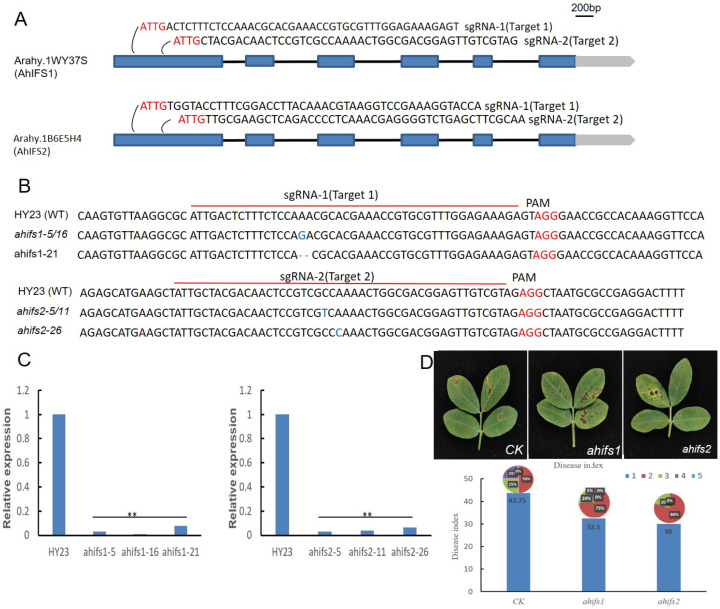
The positioning of target sites on the *AhIFS1* and *AhIFS2* genes, along with the identification of mutant lines generated through the CRISPR/Cas9 system, is presented. (**A**) The CRISPR/Cas9 sgRNA-1 and sgRNA-2 specifically target the first exon of both *AhIFS1* and *AhIFS2* genes. The sequence ATTG serves as the initial recognition motif, while the protospacer adjacent motif (PAM) is highlighted in red. Gray boxes denote untranslated regions (UTRs), blue boxes represent exons, and black lines indicate introns. (**B**) The edited sequences at the target site are illustrated for HY23 and various mutant lines. Blue nucleotides signify single nucleotide polymorphisms (SNPs) found in mutants *ahifs1*–*5/16*, *ahifs2*–*5/11*, *ahifs2*–*26*; additionally, a blue ‘–’ indicates deletions observed between HY23 and mutant *ahifs1-21*. The target sequence is indicated by a red underline, with nucleotides marked in red representing PAM. (**C**) Relative expression of *AhIFS* genes in the mutant. ** *p* < 0.01; Dunnett’s test. (**D**) The symptoms and disease index of peanut mutant leaves inoculated with *P. arachidicola* conidial suspensions at 14 dpi.

To further understand the function of AhIFSs, we designed sgRNAs to edit the two homologous *AhIFS* genes using CRISPR/Cas9 technology. Two independent CRISPR/Cas9 vectors, pCAMBIA1300-cas9-AhIFS1 and pCAMBIA1300-cas9-AhIFS2, were created and individually introduced into the ‘huayu23’ (HY23) peanut cultivar by pollen tube injection instead of going through rooting and acclimatization. These plants were then allowed to grow and produce putative transgenic plants for this experiment. A total of 68 and 73 independent positive transgenic lines were obtained from the *∆ahifs1* and *∆ahifs2* transformant events, respectively. Leaves of the knockout mutant plants were spray-inoculated with conidial suspensions of *P. arachidicola* and their phenotypes were observed. Necrosis symptoms were visually observed at 14 days post-inoculation (dpi), and Figure 6D shows that the lesion diameters and disease index of the mutant peanut leaves were significantly larger than those of the controls.

### 2.9. Detection of Putative Cis-Elements Using Promotor Region Analysis

**Figure 7:**
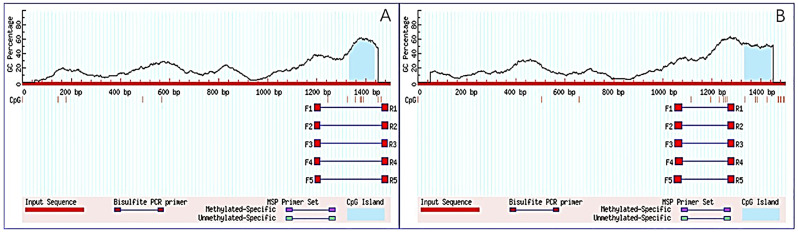
Prediction of CpG island in *AhIF* genes promoter (**A**) *AhIFS1* gene; (**B**) *AhIFS2* gene.

An analysis of the cis-acting elements in the promoter region of these two Ah*IFS* genes, showed that there were abundant cis-acting elements in the promoters. The cis-element predictions indicated that Box 4 and the G-box were light responsive elements and that STRE is a cis-element involved in stress responses. The presence of hormone related cis-elements, such as the TGACG-motif, ERE, ABRE, and the TCA element, in the promoter region indicates how responsive the AhIFSs were to MeJA, ethylene, abscisic acid, and salicylic acid (Table S3). The CpG island may play important roles in gene expression regulation and mutation. There was a CpG island in the promoter region of the AhIFS genes (Figure 7), the length of the island was more than 100 bp, and the GC content was more than 50%. It is also possible that there might be transcription regulation binding sites in this region, which future studies could use to investigate the environmental response and regulatory mechanism associated with the AhIFSs promoter.

## 3. Discussion and Conclusions

Peanut web blotch can manifest throughout the entire peanut growing season, typically resulting in yield losses of 10–20%, which may exceed 30% in severe cases [7]. Breeding for disease-resistant varieties represents the most effective strategy for managing plant diseases. However, this necessitates a comprehensive analysis of the interaction between peanuts and *P. arachidicola* to identify genes associated with disease resistance. In this study, transcriptome analysis of the interaction between peanuts and *P. arachidicola* revealed that 2989 common genes (779 up-regulated and 2210 down-regulated) were differentially expressed in peanut leaves infected with *P. arachidicola* at 7, 14, and 21 days compared to controls. Through transcriptomic analysis during responses to *Fusarium oxysporum* [20] infection and drought stress, a total of 3746 and 1629 differentially expressed genes (DEGs) were identified respectively. Thus, our transcriptome data are reliable and enhance understanding of candidate genes involved in the resistance pathway against *P. arachidicola*.

Gene ontology and KEGG analyses were employed to screen DEGs for functional genes responsive to *P. arachidicola* infection, and results indicated that Ah*IFS* genes exhibited significant enrichment within these pathways. Notably, AhIFS1 and AhIFS2 showed strong induction following infection by *P. arachidicola*. Overexpression of these two genes enhanced resistance levels against *P. parasitica* in *N. benthamiana* leaves while knockout experiments diminished disease resistance against *P. arachidicola* in peanuts—collectively demonstrating that AhIFSs may play pivotal roles in conferring peanut resistance to this pathogen.

Flavonoids and isoflavonoids are crucial secondary metabolites involved not only in plant growth and development but also as phytoalexins defending against biotic and abiotic stresses [21]. Numerous studies have established that genetic engineering can effectively increase isoflavone content across both legumes and non-legumes. For instance, overexpression of key enzyme-coding genes such as GmCHI, GmCHR, or GmUGT has been shown to elevate isoflavone levels significantly within soybeans [22,23,24]. Additionally, introducing an IFS-like cytochrome *P450* gene from italics *Elaeis guineensis* into italics *Pueraria mirifica* in Thailand resulted in substantial increases in daidzein and genistein [25]. Furthermore, Pokhrel et al. [26] (2021) successfully introduced soybean isoflavone synthase Gm-IFS1 into rice plants where transgenic lines displayed detectable dye lignin production alongside certain resistances to rice blast.

Consequently, the investigation into flavonoid biosynthesis remains critical research focus due not only to its implications for human nutrition but also its potential health benefits—including cancer prevention as well as cardiovascular protection attributed specifically to soybean-derived isoflavones [27]. Within plants, isoflavonoids serve as signaling molecules inducing nodule formation-related gene expression among legumes [28]. While their primary function involves stimulating nod factor production by rhizobia during legume nodulation, they additionally regulate other essential symbiotic responses including alterations related to growth, motility, P acquisition, and Fe uptake among others [29]. Moreover, isoflavones contribute significantly towards microbial interactions acting defensively against pathogen infections through phytoalexin activity [30,31]. Interestingly, isoflavonoids predominantly arise from leguminous species—with *Glycine max* and *Trifolium pretense* being recognized as leading sources regarding their content. In addition, such compounds exist albeit minimally across various families including *Medicago sativa*, *Lotus corniculatus*, *Cicer arietinum*, *Pueraria lobata*, *Lupinus micranthus*, and other beans [32]. However, little information currently elucidates why specific plants produce these compounds nor how they regulate them.

Isoflavone synthase constitutes one key enzyme within the biosynthetic pathway dedicated toward producing isoflavones [33]. The flavonoids and isoflavones biosynthesis pathways facilitate antitoxin generation while functioning as signal molecules between florae & microorganisms. Isoflavone synthesis initiates via phenylalanine deamination catalyzed by phenylalanine ammonia lyase (PAL), with subsequent substrate processing directed toward generating final products inclusive their derivatives. The findings obtained substantiate an integral role played by isoflavonoids concerning both vegetative growth and abiotic stress. Resilience was demonstrated by mutant observations (Figure S1), which showed clear signs of regrowth delay and early aging symptoms. Previous investigations indicate heightened levels observed under stress conditions affecting *L. japonicus* WT specimens [34,35].

The isoflavonoid biosynthesis genes are regulated by transcription factors from different families, e.g., R2R3-MYB transcription factors and basic helix-loop-helix, and WD40 proteins [36,37]. The results show that there were abundant cis-acting elements in the promoters of the two *AhIFS* genes. Box 4 and the G-box are light responsiveness elements and STRE is a cis-element that is involved in stress responses. The presence of hormone related cis-elements such as the TGACG-motif and the ERE, ABRE, and TCA-elements in the promoter region, indicate that *AhIFS* genes respond to MeJA, ethylene, abscisic acid, and salicylic acid. A promoter analysis of the two *AhIFS* genes showed that several defense-related cis-elements were distributed in the promoter region. In summary, these two *AhIFS* genes are regulated by transcription factors and participate in the bio-synthesis of isoflavones to regulate peanut resistance.

CRISPR/Cas9-mediated gene editing has opened an entirely new field for genetic improvement in plant genomics and biotechnology. This system has worked effectively in many crop species and has been found to be a valuable tool for gene functional studies and crop improvement. This system has worked effectively in many crop species and has been found to be a valuable tool for gene functional studies and crop improvement. In this study, it provided a rapid and efficient method for the targeted and inheritable mutagenesis of peanut genes. CRISPR/Cas9-mediated editing in peanuts could lead to significant advancements in nutritional quality, disease resistance, abiotic stress tolerance, and improved health aspects, such as allergenicity and oil quality. Despite the obvious potentials of CRISPR/Cas9 in plant breeding, there are ongoing concerns and debates regarding its precision targeting and potential unintended effects at off-target genomic sites [38,39], which can result in mosaic mutations [40]. Additionally, the high efficiency of targeted mutations may result from pollen tube transformation, which is particularly effective for peanut transformation [41]. These results demonstrated that the combined use of pollen tube transformation (PTT) and the CRISPR/Cas9 system offers an efficient system for editing the allotetraploid peanut genome. This approach will be valuable for functional gene analysis in this important crop species.

Although this study provides valuable molecular information about the peanut responses to *P. arachidicola* infection, the key genes regulating flavonoid biosynthesis and their disease resistance functions need to be further explored and verified. Furthermore, the two *AhIFSs* have differences in protein sequences, they have similar transcriptional patterns and similar functions in regulating disease resistance response. There may be some differences in other aspects, which we have not yet studied. The soybean isoflavones determination is using HPLC and HPLC-MS technology [42]. The *AhIFS* gene is involved in the synthesis of isoflavones, and the isoflavone content of the gene knockout mutants were not determined. We will refer to this method for further study in the future. What’s more, we can screen and identify transcription factors binding the *AhIFS* genes promoter region. The expression of this transcription factor was also induced by *P. arachidicola* infection. By studying the expression of transcription factors and the transcriptional activation activity of isoflavone synthesizing genes, the molecular mechanism of regulating the expression level of AhIFS protein by transcription factors was analyzed to better understand how to enhance the resistance of peanut to *P. arachidicola* infection. The analyses performed in this study demonstrated that a gene network responds to *P. arachidicola* infection and the results provided novel insights to the biological functions of isoflavone biosynthesis genes.

## 4. Materials and Methods

### 4.1. Plants and Strains

The susceptible variety Luhua No. 8 was used for infection test, and high-conversion variety Huayu No. 23 was used for genetic transformation to obtain transgenic plants. All the seeds were provided by the Shandong Peanut Research Institute Seed Resource Bank, China. The greenhouse experiment was conducted at the Shandong Peanut Research Institute, Qingdao, China, and the temperature in the greenhouse was 26~28 °C. Healthy peanut seeds were soaked in 75% alcohol for 0.5–1 min, immersed in sodium hypochlorite for 15 min, and washed three times with sterilized water. Then, three seeds were sown in 22-cm pots (sterilized field soil) and the pots were placed in an artificial climate chamber with a 14-h light and 10-h dark photocycle. *N. benthamiana* plants were grown in an environmentally controlled growth chamber at 25 °C and 70% humidity with a photoperiod of 16 h and a dark cycle of 8 h. *P. arachidicola* isolates from peanut plants with typical web blotch symptoms were used in this study. *P. arachidicola* came from Dou Daolong Laboratory of Nanjing Agricultural University.

### 4.2. RNA Sequence (RNA-Seq) Analysis

Peanut detached leaves that were 8 weeks old were inoculated with suspensions containing 1 × 10^6^ conidia/mL *P. arachidicola* conidia. The leaves were collected and examined at 0, 7, 14, and 21 dpi and then subjected to an RNA-seq analysis. The plant material was harvested and immediately frozen in liquid nitrogen. Total RNA was then extracted using Trizol reagent (Tiangen Biotechnology Co., Ltd., Beijing, China). To eliminate any genomic DNA contamination, the RNA was treated with DNase I at 37 °C for 30 min. The RNA from three biological replicates of each sample was pooled and sequenced on the Illumina HiSeq 2000 platform (Illumina, San Diego, CA, USA). All clean reads were mapped to the *A. hypogaea* reference gene sequences [43] (https://data.legumeinfo.org/Arachis/hypogaea/genomes/Tifrunner.gnm1.KYV3/) using the TopHat (version 2.1.1) sequence analysis tool with default parameters [44]. Gene expression levels were normalized as fragments per kilobase (kb) of exon per million fragments mapped (FPKM), based on the length of each gene and the read count mapped to that gene, utilizing the Cufflinks -2.0.2 tool [45]. Functional annotation of the genes was performed using a BLAST analysis (https://blast.ncbi.nlm.nih.gov/Blast.cgi/ accessed on 15 July 2020) to query the sequences against the NCBI non-redundant database, applying an E-value cut-off of 10^−5^.

### 4.3. Analysis of the DEGs

Reads were calculated for each single gene using HTSeq v0.6.1 [46], and gene expression levels were calculated as FPKM based on gene length and read counts [47]. Differential expression analysis was performed for each sample using the DEG-seq R package (Bioconductor version: 3.18)and *p* values were adjusted using q-values [48], which was set as the threshold for significant differential expression. The DEGs were defined as genes with more than a 2-fold difference in expression and q ≤ 0.05.

### 4.4. GO and KEGG Enrichment Analyses

The GO enrichment analysis of differentially abundant transcripts (DATs) was performed by the GO-seq R software package (Bioconductor version: 3.18), which is based on the Wallenius non-central hypergeometric distribution [49] that adjusts for gene length bias in DATs. The KEGG database [50] can be used to leverage information at the molecular level, in particular genome sequencing and other high-throughput experimental techniques (https://www.genome.jp/kegg/ accessed on 1 June 2020) to generate large-scale molecular datasets to determine the high-level function and utility of biological systems (e.g., cells, organisms, and ecosystems).

### 4.5. Phylogenetic Tree Analysis of the AhIFS Orthologs

The IFS protein sequences were been analysis in NCBI Blast (https://blast.ncbi.nlm.nih.gov/Blast.cgi) website, found a large number of homologous proteins, show that the protein is conservative in different species. We selected homologous sequences of 11 different species, and ran these 12 protein sequences in MEGA6.0 software to construct phylogenetic tree according to Maximum Likelihood method. Multiple sequence alignment was performed by Clustal W, and the phylogenetic tree was constructed using MEGA5.0 software from results that had been aligned by the neighbor-joining (NJ) method in MEGA7 software version 7.0.2.6. The protein domain and motif analyses were performed using the conserved domain database (http://smart.embl-heidelberg.de/) and the IFS protein sequences and 11 homologous sequences were downloaded from the National Center for Biotechnology Information (NCBI). The protein sequences of the putative IFS proteins in were aligned using the Clustal X program (version 1.83) with the default parameters.

### 4.6. Quantitative PCR Analysis

Total RNA extraction, cDNA synthesis, and qRT-PCR assay were performed according to the method of Yu et al. [51]. Briefly, total RNA was extracted from 0.1 g of fresh tissue using Trizol reagent (Tiangen Biotechnology Co., Ltd.), and the quality and integrity of RNA was detected using Nanodrop 2000 and 0.8% agarose gel. A 20 μL aliquot of cDNA was diluted to 60 μL with water, and 2 μL of the diluted cDNA was used for analysis. For qRT-PCR analysis, primers specific for the *AhIFS* gene were designed and synthesized by GenScript (Nanjing, China) (product size 100~200 bp; Tm 55~60 °C). All reactions were performed on an Icycleri Q5 system (Bio-Rad, Hercules, CA, USA) using the SYBR Green Supermix kit (Vazyme Biotech, Nanjing, China), and the expression levels of the AhIFS gene and relative expression levels were calculated as 2^−ΔCT^ values. Quantitative PCR reactions were performed using at least three biological replicates.

To analyze the response of AhIFS1 and AhIFS2 expression to GSH, we removed 6 healthy and fully unfolded upper leaves from 30-day-old peanut plants and immediately immersed them in 100 mL of GSH preparation (10 mM, pH 5.8) containing 0.005% Silwet, which could help to reduce leaves surface tension. Leaves at the same location of the control plant were also isolated and soaked in a glutathione-free solution with a pH of 5.8. The controlled and treated leaves were gently shaken in the dark (100 rpm) in an oven-controlled crystal oscillator at 25 °C. Samples were collected by filtration at 4 time points (0, 3, 6, and 9 h after incubation) [19,52].

### 4.7. Phytophthora Infection Assays

The cDNA fragments of AhIFSs were cloned into pBINPLUS with a HA-tag [53]. *Agrobacterium* GV3101 with *AhIFS* genes were transformed by electroporation and infiltration of *Agrobacterium* suspensions [54]. The overnight medium was diluted 10 times in LB/kanamycin/rifampicin and cultured to OD600 0.6~0.8. The cells were harvested by centrifugation and resuscitated with reaction liquid (10 mM MES-NaOH, pH 5.6 and 10 mM MgCl_2_). Adjust the suspension to OD600 0.6 and add acetyl syringone to 150 μM. The bacterial suspension was incubated at 28 °C for 2–3 h in dark place, and then infiltrated into the leaves of 5 week-old *N. benthamiana* leaves with a needle-free syringe [55]. *A. tummefacens* carrying *AhIFS* genes were prepared according to the description of Kamoun et al. (2003) [56]. The leaves with high *AhIFS* genes expression were removed and parasitical tests were performed. Lesion diameter was measured at 36 and 48 hpi. Significant differences were identified using the *t*-test. Mycelium extension in host cells is observed using ultraviolet light to assess pathogen development [57].

### 4.8. CRISPR/Cas9 Plasmid Construction and Peanut Transformation

The targets were designed using the online tool CRISPR-GE (http://skl.scau.edu.cn/primerdesign/ accessed on 1 June 2020). In this study, we utilized CRISPR/Cas9-related vectors, including a CRISPR/Cas9 binary vector. Two single-guide RNA (sgRNA) target sequences were designed and incorporated into the sgRNA expression cassette psgR-Cas9-At. The sgRNA sequences were then cloned into the pCAMBIA1300 vector, thus completing the pCAMBIA1300-cas9_AhIFSs construct. Subsequently, the the constructed vectors were introduced into *A. tumefaciens* strain GV3101. The overnight medium was diluted 10-fold in LB medium supplemented with kanamycin and rifampicin, then cultured to an OD600 of 0.6~0.8. The cells were harvested by centrifugation and resuspended in a solution of 10 mM MES-NaOH (pH 5.6) and 10 mM MgCl_2_ prior to further analysis. The suspension should be adjusted to an OD600 of 0.6, and acetyl syringone was added to a final concentration of 150 μM. The bacterial suspension was incubated at 28 °C for 2 to 3 h before being introduced into the blooming peanut pollen tube.

### 4.9. Mutant Identification

Genomic DNA was isolated from both the HY23 and transgenic plants using hexadecyl trimethyl ammonium bromide (CTAB) method. Specific PCR primers targeting the *Cas9* gene were designed to identify the transgenic plants (Cas9-F: 5′-GGTTCTGTCAGTTCCAAACG-3′, Cas9-R: 5′-CGTCACCTTCTCCGTCGAAC-3′). Additionally, specific primers in the flanking region of the CRISPR/Cas9 targets were used for PCR amplification to analyze the targeted mutagenesis. (IFS1-F: 5′-ATGCCCACCGTAGTCGCCTCTT-3′, IFS1-R: 5′-TTACGAGGAAAGGAGCTTTG-3′; IFS2-F: 5′-ATGGTACCTTTCGGACCTTAC-3′, IFS2-R: 5′-TTACGAGGAAAGGAGCTTTG-3′). The PCR products were either directly sequenced or cloned into pMD18-T (Takara, Shiga, Japan) and subsequently subjected to Sanger sequencing.

### 4.10. Mutant Phenotype Analysis

The mutant phenotypes were analyzed using in vitro leaf inoculation. A total of 20 leaves showing good growth were selected and sprayed with an inoculum suspension of 1 × 10^6^ conidia/mL, which was prepared according to Zhang [58]. Two weeks post-inoculation, 20 inoculated leaves from the main stem were collected, and the lesion area on each was scanned. The indoor classification standard for peanut web blotch is as follows: scale 0, no lesion detected; scale 1, 0 < lesion area < 10%; scale 2, 10% ≤ lesion area < 25%; scale 3, 25% ≤ lesion area < 50%; and scale 4, 50% ≤ lesion area [59].

### 4.11. Promoter Analysis

The predicted cis-regulatory elements, which are the promoter sequences (1500 bp upstream of the transcription start site) for the two AhIFS genes in peanut, were identified using the PlantCARE database. Subsequently, CpG island prediction software, MethPrimer (http://www.urogene.org/methprimer/ accessed on 1 June 2020), utilising the default settings (island size > 100 bp, GC percentage > 50%, and an observed/expected CpG ratio > 0.6), was employed to identify CpG islands within the *AhIFS* genes region [60].

### 4.12. Statistical Analysis

The data are presented as means ± standard deviations (SD) derived from at least three biological replicates. The presence of different letters indicates statistically significant differences, as determined through one-way analysis of variance (ANOVA) and Duncan’s multiple range test. The analyses were conducted using SAS (version 8.1, SAS Institute, Cary, NC, USA) at a significance level of *p* < 0.05, and asterisks represent statistical significances according to the student’s *t*-test at a significance level of *p* < 0.05 (*) or 0.01 (**).

## Data Availability

The data and materials that support the findings of this study are available from the corresponding author upon request.

## Supplementary Materials

The following supporting information can be downloaded at: https://www.mdpi.com/article/10.3390/plants13212948/s1, Figure S1. The transgenic peanut lines; Table S1: Novel Transcript Summary; Table S2: GO enrichment analysis; Table S3: Cis-element predictions for the *AhIFS* genes.

## Abbreviations

KEGG	Kyoto Encyclopedia of Genes and Genomes
ORFs	open reading frames
QTL	quantitative trait locus
PAL	phenylalanine ammonia lyase
CHS	chalcone synthase
IFS	Isoflavone synthase
F3H	flavanone-3-hydroxylase
DEG	differentially expressed genes
BP	biological process
CC	cellular component
MF	molecular function

## References

[1] Jiang L., Hua D., Wang Z., Xu S. (2010). Aqueous enzymatic extraction of peanut oil and protein hydrolysates. Food Bioprod. Process.

[2] Hao K., Wang F., Nong X., Mcneill M.R., Liu S., Wang G., Cao G., Zhang Z. (2017). Response of peanut *Arachis hypogaea* roots to the presence of beneficial and pathogenic fungi by transcriptome analysis. Sci. Rep..

[3] Pettit R.E., Philley G.L., Smith D.H., Taber R.A. (1986). Peanut Web Blotch: II Symptoms and Host Range of Pathogen. Peanut Sci..

[4] Manamgoda D.S., Rossman A.Y., Castlebury L.A., Crous P.W., Madrid H., Chukeatirote E., Hyde K.D. (2014). The genus Bipolaris. Stud. Mycol..

[5] Taber R.A., Pettit R.E., Philley G.L. (1984). Peanut Web Blotch: I. Cultural Characteristics and Identity of Causal Fungus. Peanut Sci..

[6] Pereira F.A., Moraes S.A.D., Franco G.A.A., Montenegro V.J.F., Antonio V.N. (2009). Characterization of rust, early and late leaf spot resistance in wild and cultivated peanut germplasm. Scientia Agri..

[7] Zhou R.-J., Cui J.-C., Fu J.-F., Xu Z., Xue C.-Y. (2015). Effect of Mixed Infection of Cercospora arachidicola and *Phoma arachidicola* on their Infection Probability and Latent Periods. Sci. Agric. Sin..

[8] Zhang L. (2019). Study on Physiological and Biochemical Resistance of Peanut (*Arachis hypogaea* L.) against Web Blotch. Master Thesis.

[9] Zhang X.Y. (2011). Inheritance of Main Traits Related to Yield, Quality and Disease Resistance and Their QTLs Mapping in Peanut (*Arachis hypogaea* L.). Ph.D. Dissertation.

[10] Mikunthan G. (1997). First report of web blotch of peanut caused by *Phoma arachidicola* in the dry zone of Sri Lanka. Plant Dis..

[11] Yu O. (2000). Production of the Isoflavones Genistein and Daidzein in Non-Legume Dicot and Monocot Tissues. Plant Physiol..

[12] Pueppke S.G. (1996). The genetic and biochemical basis for nodulation of legumes by rhizobia. Crit. Rev. Biotechnol..

[13] Rhijn P.V.V., Vanderleyden J. (1995). The Rhizobium-plant symbiosis. Microbiol. Rev..

[14] Rivera-Vargas L.I., Schmitthenner A.F., Graham T.L. (1993). Soybean flavonoid effects on and metabolism by *Phytophthora sojae*. Phytochemistry.

[15] Grotewold E., Sainz M.B., Tagliani L., Hernandez J.M., Bowen B., Chandler V.L. (2000). Identification of the residues in the Myb domain of maize C1 that specify the interaction with the bHLH cofactor R. Proc. Natl. Acad. Sci USA.

[16] Ramsay N.A., Glover B.J. (2005). MYB-bHLH-WD40 protein complex and the evolution of cellular diversity. Trends Plant Sci..

[17] Yi J., Derynck M.R., Li X., Telmer P., Marsolais F., Dhaubhadel S. (2010). A single-repeat MYB transcription factor, GmMYB176, regulates CHS8 gene expression and affects isoflavonoid biosynthesis in soybean. Plant J..

[18] Yu O., Shi J., Hession A.O., Maxwell C.A., Mcgonigle B., Odell J.T. (2003). Metabolic engineering to increase isoflavone biosynthesis in soybean seed. Phytochemistry.

[19] Robbins M.P., Hartnoll J., Morris P. (1991). Phenylpropanoid defence responses in transgenic Lotus corniculatus 1. Glutathione elicitation of isoflavan phytoalexins in transformed root cultures. Plant Cell Rep..

[20] Wang M., Zhu L., Zhang C., Zhou H., Tang Y., Cao S., Chen J., Zhang J. (2024). Transcriptomic-Proteomic Analysis Revealed the Regulatory Mechanism of Peanut in Response to *Fusarium oxysporum*. Int. J. Mol. Sci..

[21] García-Calderón M., Pérez-Delgado C.M., Palove-Balang P., Betti M., Márquez A.J. (2020). Flavonoids and Isoflavonoids Biosynthesis in the Model Legume Lotus japonicus; Connections to Nitrogen Metabolism and Photorespiration. Plants.

[22] Wu N., Wang P.-W., Lin N., Lu S., Feng Y.-Q., Rong J., Zhang Z., Qu J. (2017). Construction of a chalcone reductase expression vector and transformation of soybean plants. Mol. Med. Rep..

[23] Yin Y.-C., Zhang X.-D., Gao Z.-Q., Hu T., Liu Y. (2019). The research progress of chalcone isomerase (CHI) in plants. Mol. Biotechnol..

[24] Nguyen H.Q., Le T.H.T., Nguyen T.N.L., Nguyen T.G., Sy D.T., Tu Q.T., Le V.S., Chu H.M., Vu T.K.L. (2020). Overexpressing GmCHI1A increases the isoflavone content of transgenic soybean (*Glycine max* (L.) Merr.) seeds. In Vitro Cell. Dev. Biol. Plant.

[25] Xu L., Kaopong R., Nualkaew S., Chullasara A., Phongdara A. (2017). Expression and functional analysis of a transgenic cytochrome P450 monooxygenase in *Pueraria mirifica*. Sains Malays..

[26] Pokhrel S., Ponniah S.K., Jia Y., Yu O., Manoharan M. (2021). Transgenic rice expressing isoflavone synthase gene from soybean shows resistance against blast fungus (*Magnaporthe oryzae*). Plant Dis..

[27] Chu S., Wang J., Zhu Y., Liu S., Zhou X., Zhang H., Wang C.-E., Yang W., Tian Z., Cheng H. (2017). An R2R3-type MYB transcription factor, GmMYB29, regulates isoflavone biosynthesis in soybean. PLoS Genet..

[28] Cheng Q., Li N., Dong L., Zhang D., Fan S., Jiang L., Wang X., Xu P., Zhang S. (2015). Overexpression of Soybean Isoflavone Reductase (GmIFR) Enhances Resistance to *Phytophthora sojae* in Soybean. Front. Plant Sci..

[29] Liu C.W., Murray J. (2016). The Role of Flavonoids in Nodulation Host-Range Specificity: An Update. Plants.

[30] Aoki T., Akashi T., Ayabe S. (2000). Flavonoids of leguminous plants: Structure, biological activity, and biosynthesis. J. Plant Res..

[31] Dixon R.A., Achnine L., Kota P., Liu C.J., Reddy M.S.S., Wang L.J. (2002). The phenylpropanoid pathway and plant defence a genomics perspective. Mol. Plant Pathol..

[32] Kaufman P.B., Duke J.A., Brielmann H., Boik J., Hoyt J.E. (1997). A comparative survey of leguminous plants as sources of the isoflavones, genistein and daidzein: Implications for human nutrition and health. J. Altern. Complement. Med..

[33] Jung W., Yu O., Lau S.M., O’Keefe D.P., Odell J., Fader G., McGonigle B. (2000). Identification and expression of isoflavone synthase, the key enzyme for biosynthesis of isoflavones in legumes. Nat. Biotechnol..

[34] García-Calderón M., Pons-Ferrer T., Mrazova A., Pal’ove-Balang P., Vilkova M., Pérez-Delgado C.M., Vega J.M., Eliášová A., Repčák M., Márquez A.J. (2015). Modulation of phenolic metabolism under stress conditions in a Lotus japonicus mutant lacking plastidic glutamine synthetase. Front. Plant Sci..

[35] Kaducová M., Monje-Rueda M.D., García-Calderón M., Pérez-Delgado C.M., Eliášová A., Gajdošová S., Petruľová V., Betti M., Márquez A.J., Paľove-Balang P. (2019). Induction of isoflavonoid biosynthesis in Lotus japonicus after UV-B irradiation. J. Plant Physiol..

[36] Grotewold E. (2005). Plant metabolic diversity: A regulatory perspective. Trends Plant Sci..

[37] Petroni K., Tonelli C. (2011). Recent advances on the regulation of anthocyanin synthesis in reproductive organs. Plant Sci..

[38] Eckerstorfer M.F., Engelhard M., Heissenberger A., Simon S., Teichmann H. (2019). Plants developed by new genetic modification techniques—Comparison of existing regulatory frameworks in the EU and non-EU countries. Front. Bioeng. Biotechnol..

[39] Manghwar H., Li B., Ding X., Hussain A., Lindsey K., Zhang X.L., Jin S.X. (2020). CRISPR/Cas systems in genome editing: Methodologies and tools for sgRNA design, of-target evaluation, and strategies to mitigate of-target efects. Adv. Sci..

[40] Mehravar M., Shirazi A., Nazari M., Banan M. (2019). Mosaicism in CRISPR/Cas9-mediated genome editing. Dev. Biol..

[41] Li A., Zhou M., Liao G., Li X., Wang A., Xiao D., He L., Zhan J. (2023). CRISPR/Cas9 gene editing in peanut by Agrobacterium tumefaciens-mediated pollen tube transformation. Plant Cell Tissue Organ Cult..

[42] Wu Q., Wang M., Simon J.E. (2003). Determination of isoflavones in red clover and related species by high-performance liquid chromatography combined with ultraviolet and mass spectrometric detection. J. Chromatogr. A.

[43] Zhuang W., Chen H., Yang M., Wang J., Pandey M.K., Zhang C., Chang W.-C., Zhang L., Zhang X., Tang R. (2019). The genome of cultivated peanut provides insight into legume karyotypes, polyploid evolution and crop domestication. Nat. Genet..

[44] Trapnell C., Pachter L., Salzberg S.L. (2009). TopHat: Discovering splice junctions with RNA-Seq. Bioinformatics.

[45] Trapnell C., Williams B.A., Pertea G., Mortazavi A., Kwan G., Baren M.J.V., Salzberg S.L., Wold B.J., Pachter L. (2010). Transcript assembly and quantification by RNA-Seq reveals unannotated transcripts and isoform switching during cell differentiation. Nat. Biotechnol..

[46] Anders S., Pyl P.T., Huber W. (2015). HTSeq-a Python framework to work with high-throughput sequencing data. Bioinformatics.

[47] Huber A.W. (2010). Differential expression analysis for sequence count data. Genome Biol..

[48] Storey J.D. (2003). The Positive False Discovery Rate: A Bayesian Interpretation and the Q-value. Ann. Stats.

[49] Young M.D., Wakefield M.J., Smyth G.K., Oshlack A. (2010). Gene ontology analysis for RNA-seq: Accounting for selection bias. Genome Biol..

[50] Kanehisa M., Araki M., Goto S., Hattori M., Hirakawa M., Itoh M., Katayama T., Kawashima S., Okuda S., Tokimatsu T. (2008). KEGG for linking genomes to life and the environment. Nucleic Acids Res..

[51] Yu J., Ai G., Shen D., Chai C., Jia Y., Liu W., Dou D. (2019). Bioinformatical analysis and prediction of *Nicotiana benthamiana* bHLH transcription factors in *Phytophthora parasitica* resistance. Genomics.

[52] Shelton D., Stranne M., Mikkelsen L., Pakseresht N., Welham T., Hiraka H., Tabata S., Sato S., Paquette S., Wang T.L. (2012). Transcription factors of Lotus: Regulation of isoflavonoid biosynthesis requires coordinated changes in transcription factor activity. Plant Physiol..

[53] Song T., Ma Z., Shen D., Li Q., Li W., Su L., Ye T., Zhang M., Wang Y., Dou D. (2015). An oomycete CRN effector reprograms expression of plant HSP genes by targeting their promoters. PLoS Pathog..

[54] Asai S., Ohta K., Yoshioka H. (2008). MAPK signaling regulates nitric oxide and NADPH oxidase-dependent oxidative bursts in *Nicotiana benthamiana*. Plant Cell.

[55] Yoshioka H., Numata N., Nakajima K., Katou S., Kawakita K., Rowland O., Jones J.D.G., Doke N. (2003). Nicotiana benthamiana gp91 phoxhomologs Nbrboh A and Nbrboh B participate in H_2_O_2_ accumulation and resistance to *Phytophthora infestans*. Plant Cell.

[56] Kamoun S., Hamada W., Huitema E. (2003). Agrosuppression: A bioassay for the hypersensitive response suited to high-throughput screening. Mol. Plant Microbe Interact..

[57] Ma J., Sun S., Whelan J., Shou H. (2021). CRISPR/Cas9-Mediated Knockout of GmFATB1 Significantly Reduced the Amount of Saturated Fatty Acids in Soybean Seeds. Int. J. Mol. Sci..

[58] Zhang X., Xu M.L., Wu J.X., Dong W.B., Chen D.X., Wang L., Chi Y.C. (2019). Draft Genome Sequence of *Phoma arachidicola* Wb2 Causing Peanut Web Blotch in China. Curr. Microbiol..

[59] Liu H., Sun Z., Zhang X., Qin L., Qi F., Wang Z., Du P., Xu J., Zhang Z., Han S. (2020). QTL Mapping of Web Blotch Resistance in Peanut by High-throughput Genome-wide Sequencing. BMC Plant Biol..

[60] Li L.C., Dahiya R. (2002). MethPrimer: Designing primers for methylation PCRs. Bioinformatics.

