# Supplementary figures and images for: An Isoflavone Synthase Gene in Arachis hypogea Responds to Phoma arachidicola Infection Causing Web Blotch
Source: Plants (Basel). 2024 Oct 22;13(21):2948. doi: 10.3390/plants13212948 (PMC11547825; doi:10.3390/plants13212948)

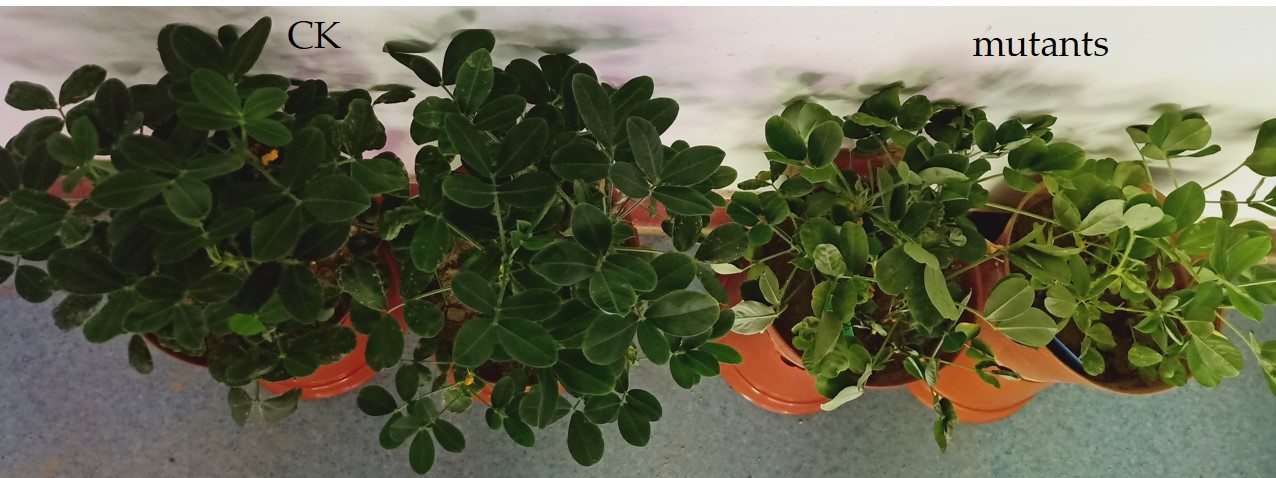

Supplement: Supplementary file 1 [file plants-13-02948-s001.zip › Figure S1.jpg]
